# A highly-selective biomimetic potassium channel

**DOI:** 10.1093/nsr/nwae242

**Published:** 2024-07-13

**Authors:** Junliang Zhu, Hu Qiu, Wanlin Guo

**Affiliations:** Key Laboratory for Intelligent Nano Materials and Devices of the Ministry of Education, State Key Laboratory of Mechanics and Control for Aerospace Structures, Institute for Frontier Science, Nanjing University of Aeronautics and Astronautics, Nanjing 210016, China; Key Laboratory for Intelligent Nano Materials and Devices of the Ministry of Education, State Key Laboratory of Mechanics and Control for Aerospace Structures, Institute for Frontier Science, Nanjing University of Aeronautics and Astronautics, Nanjing 210016, China; Key Laboratory for Intelligent Nano Materials and Devices of the Ministry of Education, State Key Laboratory of Mechanics and Control for Aerospace Structures, Institute for Frontier Science, Nanjing University of Aeronautics and Astronautics, Nanjing 210016, China

**Keywords:** biomimetic design, carbon nanotube, K^+^/Na^+^ selectivity, molecular dynamics simulations

## Abstract

Reproducing the outstanding selectivity achieved by biological ion channels in artificial channel systems can revolutionize applications ranging from membrane filtration to single-molecule sensing technologies, but achieving this goal remains a challenge. Herein, inspired by the selectivity filter structure of the KcsA potassium channel, we propose a design of biomimetic potassium nanochannels by functionalizing the wall of carbon nanotubes with an array of arranged carbonyl oxygen atoms. Our extensive molecular dynamics simulations show that the biomimetic nanochannel exhibits a high K^+^ permeation rate along with a high K^+^/Na^+^ selectivity ratio. The free energy calculations suggest that the low Na^+^ permeability is the result of the higher energy barrier for Na^+^ than K^+^ at the channel entrance and ion binding sites. In addition, reducing the number of ion binding sites leads to an increase in the permeation rate but a decrease in selectivity. These findings not only hold promise for the design of high-performance membranes but also help understand the mechanism of selective ion transport in biological ion channels.

## Teaser

This work proposes a bio-inspired design of artificial potassium channels that can reproduce the outstanding selectivity and permeability of the biological potassium channel KcsA.

## INTRODUCTION

Ion transport through nanoscale channels in cellular membranes plays a crucial role in many physiological processes required for the maintenance of life, such as the generation and propagation of nerve impulses [1–3]. Through millions of years of evolution, these biological ion channels achieve remarkably high ion selectivity while maintaining exceptional permeability [4,5]. For example, potassium channels can separate K^+^ from Na^+^ with a selectivity ratio exceeding 1000 while allowing for K^+^ conduction at a rate close to the diffusion limit [6]. However, their heightened sensitivity to environmental changes often results in a loss of bioactivity *ex vivo*, significantly limiting their usage in technical applications. Hence, it is of great interest to develop artificial analogs (namely biomimetic nanochannels) that can reproduce the key functions of these biological channels. These nanochannels may find applications in many fields such as water desalination [7,8], energy conversion [9,10] and biosensing [11,12].

Among all pairs of ions, K^+^ and Na^+^ are probably the most difficult to differentiate from each other, because they are both monovalent and similar in molecular size. Biological potassium channels accomplish this task with their selectivity filter, which contains a precisely arranged array of carbonyl oxygen atoms forming closely spaced carbonyl binding sites to coordinate ions [13–15]. Aiming to realize similar selectivity in artificial systems, many nanomaterials and nanostructures were used to construct nanochannels containing similar ion binding sites as in biological channels, including crown ethers, solid-state nanopores, foldamers, etc. [16–20]. In parallel, molecular dynamics (MD) simulations predicted that functionalized monolayer or multi-layer graphene and carbon nanotubes (CNTs) can serve as artificial potassium channels [21–23]. Recently, Li *et al.* predicted that a twisted carbonyl-oxygen-modified bi-layer graphene nanopore exhibits very high K^+^/Na^+^ selectivity [24]. However, these nanochannels still differ from the KcsA channel either in the number of binding sites or the channel diameter.

Here, we propose a novel design of artificial potassium nanochannels, based on single-walled nanotubes modified with regularly arranged carbonyl atoms. The major structural feature of the present channels is that they fully mimic the arrangement of carbonyl groups in the KcsA selectivity filter and thus have a same number of binding sites and almost identical diameter. The biomimetic nanochannel exhibits a high K^+^ permeation rate along with a high K^+^/Na^+^ selectivity ratio. These results not only enhance our understanding of ion transport in nanochannels but also are useful for designing nanochannels to sieve monovalent ions.

## RESULTS AND DISCUSSION

### Atomic model of the biomimetic nanochannel

**Figure 1.**
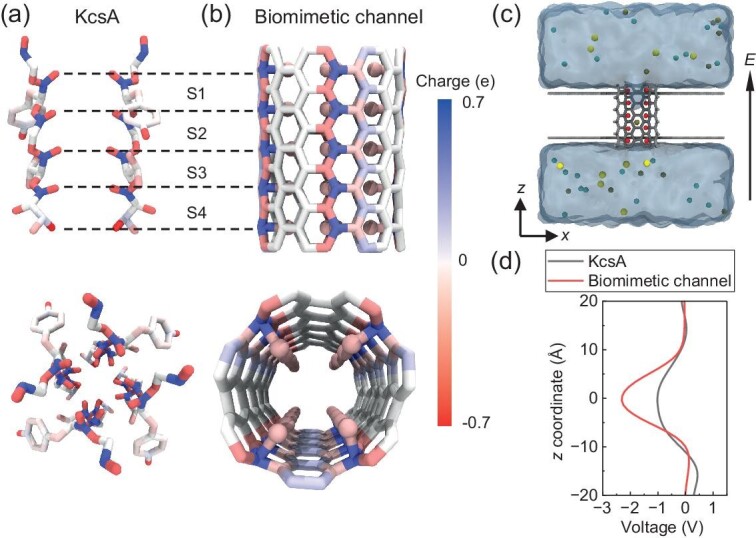
Biological and biomimetic nanochannels. (a) Side (top panel) and top views (bottom panel) of the selectivity filter of the biological KcsA channel. Four binding sites (S1, S2, S3 and S4) are aligned in series along the selectivity filter. (b) Side (top panel) and top views (bottom panel) of the biomimetic nanochannel. Twenty carbonyl oxygens (shown as spheres) were modified inside the nanotube to mimic the structure of the KcsA selectivity filter. All atoms are colored based on their charges. (c) The simulation system. The modified nanotube channel was embedded between two graphene sheets, separating the solution into two chambers. The red spheres represent the carbonyl oxygens, and the yellow, brown and blue spheres represent Na^+^, K^+^ and Cl^−^ ions, respectively. An electric field was applied along the positive direction of the *z*-axis. (d) Electrostatic potential distribution profiles inside the KcsA selectivity filter (black line) and the biomimetic nanochannel (red line).

The atomic model of the biomimetic channel was built according to the selectivity filter structure of the KcsA channel (Fig. 1a). Specifically, a 0.95 nm-diameter (7,7) single-walled CNT was used, and 20 carbonyl oxygens in four rows were modified inside the nanotube, which was inspired by the four layers of carbonyl groups in the KcsA selectivity filter (Fig. 1b). In other words, these oxygen atoms are connected to the carbon atoms on the CNT wall to form C–O bonds. Subsequently, the nanochannel was subjected to a 10% strain to ensure that the vertical distance between adjacent carbonyl layers is also comparable to that in KcsA (see detailed procedure in Methods section). The modified nanochannel was then embedded between two graphene sheets and attached to a mixed solution with a total concentration of 1 M (0.5 M KCl and 0.5 M NaCl) at both ends of the channel (Fig. 1c). The final simulation system measured 5.1 × 5.1 × 5.1 nm^3^ in dimension, containing ∼10 500 atoms (see other simulation details in Methods section and Supplementary text).

We first compared the electrostatic potential distribution profile inside the biomimetic channel with that in KcsA (Fig. 1d). As expected, these two profiles are generally similar to each other, despite the slight difference in the depth of the valley. This observation implies that the biomimetic channel has the potential to reproduce the ion selectivity of the KcsA channel.

### Ion permeation rates under different electric fields

**Figure 2.**
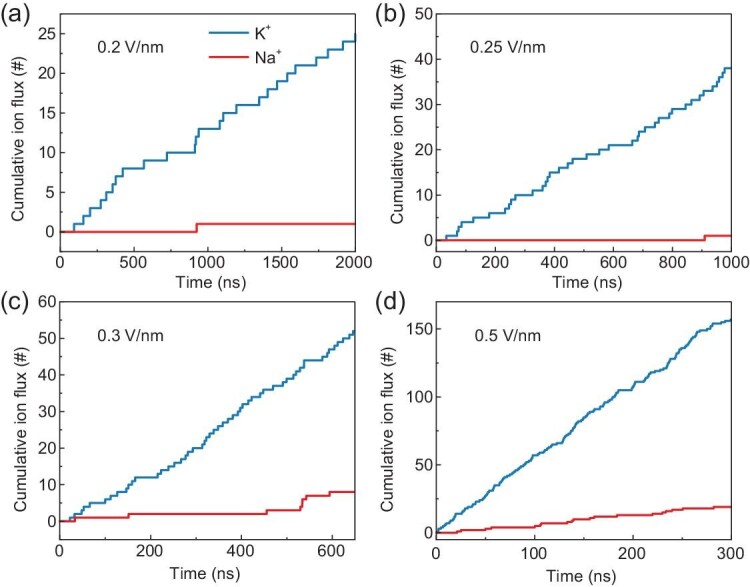
(a–d) Cumulative ion fluxes of K^+^ (blue line) and Na^+^ (red line) through the biomimetic nanochannel under different electric fields.

The permeability and selectivity of the nanochannel can be obtained by counting the permeated K^+^ and Na^+^ under various electric fields. A similar strategy was often used to evaluate the ion selectivity of various nanochannels in MD simulations [21,25–27]. As shown in Fig. 2, it is as expected to see that the permeation rates of K^+^ and Na^+^, namely the slopes of the cumulative ion flux profiles, both increase with the field (from 0.2 V/nm to 0.5 V/nm). The permeation rate of K^+^ is higher than that of Na^+^, which suggests that the biomimetic nanochannel can selectively transport K^+^ over Na^+^. Notably, under low fields of 0.2 and 0.25 V/nm, K^+^ can permeate at a rate of 1.2∼3.7 × 10^7^ #/s (Fig. 2a and b), close to that in biological potassium channels (∼10^8^ #/s); only a single Na^+^ permeates through the biomimetic nanochannel in our microsecond simulations. Further reduction in the field strength may fully block the Na^+^ permeation. These facts suggest that the biomimetic nanochannel shows exceptional selectivity of K^+^ over Na^+^. With the increase of the electric field (to 0.3 and 0.5 V/m), the Na^+^ permeation rate significantly rises, but remains 6.8 to 8.3 times lower than K^+^, suggesting that the biomimetic nanochannel remains K^+^-selective (Fig. 2c and d). However, the ion selectivity seemingly decreases with the increasing field.

In order to understand the underlying mechanism, we first examined the ion hydration properties under various electric fields. As shown in Fig. S1a and S1b in the online supplementary file, the hydration number distribution profiles at different electric fields are almost identical for a given ion (K^+^ or Na^+^). We then calculated the probability distribution of the orientation angle of water molecules inside the ions’ first hydration shells at different field strengths (Fig. S1c and S1d). It can be found that the orientation distribution profiles differ from each other under different electric fields, suggesting that the electric field can significantly reorient the ion hydration shells (though the number of water molecules therein does not change). Our previous work has revealed that such electric field-induced reorientation alters the energy barriers of ion permeation through the channel, and in turn, the ion selectivity [28].

### Ion permeation dynamics

**Figure 3.**
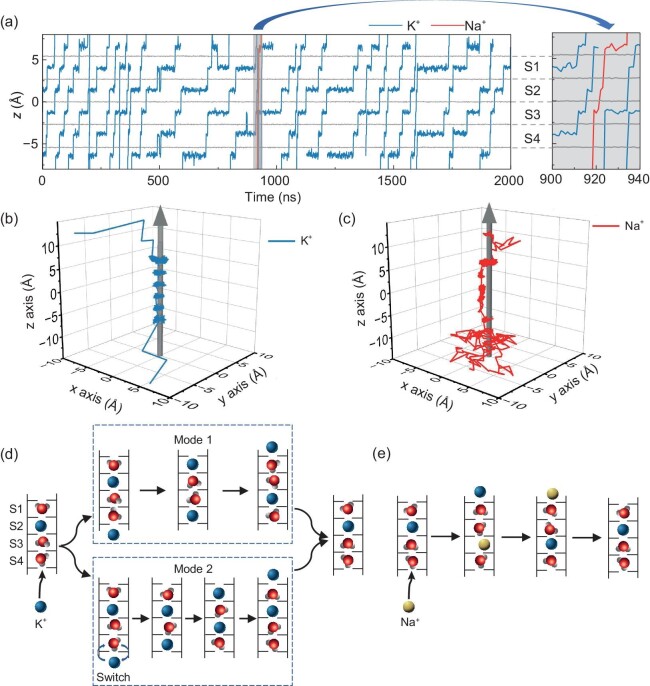
Permeation dynamics of K^+^ and Na^+^ through the nanochannel under 0.2 V/nm. (a) The *z* coordinates of ions passing through the nanochannel. Many K^+^ permeation events, shown in blue curves, are identified in the simulation trajectory. The permeation process of a single Na^+^ is identified and shown in a magnified view on the right side (red line). The grey lines represent the average position of each carbonyl ring layer. (b and c) Representative three-dimensional trajectories of K^+^ (b) and Na^+^ (c) transporting through the nanochannel. The arrows indicate the cental axis of the nanochannel. (d) Two different permeation modes of K^+^. Blue arrows in mode 2 represent the switching between an incoming K^+^ ion and the water molecule at S4. (e) The permeation mode of Na^+^.

We further monitored the kinetic process of ions passing through the biomimetic nanochannel. Given the fact that the biomimetic nanochannel shows significant K^+^ selectivity under low electric fields, we particularly focus on the situation under the electric field of 0.2 V/nm. Each binding site (S1–S4) in the nanochannel was either occupied by an ion or a water molecule, resembling the situation in the KcsA channel. Variations of the *z*-coordinates of K^+^ and Na^+^ passing through the nanochannel during the full trajectory are shown in Fig. 3a (detailed permeation dynamics with water molecules are shown in Fig. S2). It is found that K^+^ transports across the nanochannel by occupying the binding sites through stepwise movement (Fig. 3a, blue lines). In contrast, Na^+^ first jumps to the S3 site and then quickly exits the channel due to the knock-on of an incoming K^+^ (Fig. 3a, red line); this manner is different from the stepwise movement of K^+^. The three-dimensional positions of a single K^+^ (Fig. 3b) and Na^+^ (Fig. 3c) further validate our above findings. In addition, it is noteworthy that the pathway of Na^+^ along the nanochannel was off-center while that of K^+^ was on-center (discussed later).

Careful examination of all the permeation events of K^+^ suggests that there are two distinct permeation modes of K^+^. In mode 1, a fully hydrated K^+^ first approaches the entrance of the nanochannel. It further moves into site S4 and knocks off the original bound K^+^ at S2 to the downward site S1 (Fig. 3d, mode 1), resembling the knock-on mechanism observed in the potassium channels. Two water molecules are in the interval of the ions in the nanochannel in mode 1. Mode 1 is the main form of K^+^ passing through the nanochannel, accounting for ∼76% of the total events. While in mode 2, K^+^ at the entrance of the nanochannel first switches positions with the water molecule at S4, such that only one water molecule is spaced between two K^+^ (Fig. 3d, mode 2). It then remains in this configuration until the ion exits the nanochannel. This mode accounts for ∼24% of the total events. As to Na^+^, the Na^+^ in the nanochannel was easy to be knocked off by an incoming K^+^ (Fig. 3e).

### Structural properties of ions and water in the nanochannel

**Figure 4.**
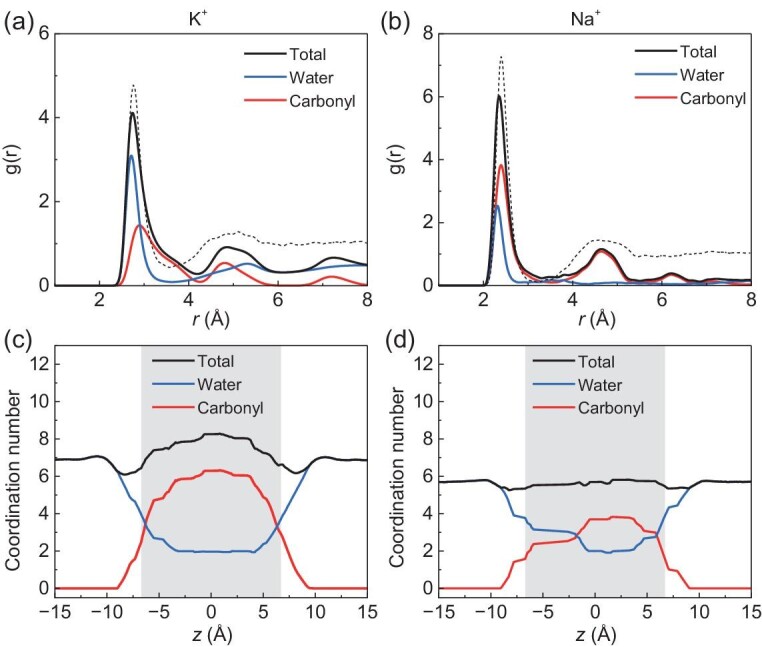
Ion coordination states in the nanochannel under 0.2 V/nm. (a and b) Ion-oxygen RDF profiles for K^+^ (a) and Na^+^ (b) with respect to water oxygens, carbonyl oxygens, and their total. The data for K^+^ and Na^+^ in bulk solution are also shown for comparison (black dashed lines). (c and d) The coordination numbers for all ligands, carbonyl oxygens only, and water oxygens only for K^+^ (c) and Na^+^ (d) when transporting through the nanochannel. The channel area is highlighted in grey.

Ion solvation has been widely recognized as a key factor governing ion transport through the nanochannel [29–31]. We then turn to understand the ion solvation properties in order to further explore the underlying selectivity mechanism. The ion-oxygen radial distribution function (RDF) profiles of K^+^ and Na^+^ in the nanochannel, with respect to carbonyl oxygen atoms (red lines), water oxygen atoms (blue lines), and their total (black lines) are shown in Fig. 4a and b, respectively. For K^+^ in the nanochannel, the first minimum of the total RDF profile (Fig. 4a, black line) and the RDF profile for the carbonyl oxygen (red line) shift noticeably to the right compared to that in bulk solution (black dashed line), from 0.36 nm to 0.42 nm. This result suggests that the distances between the K^+^ ion and the coordinated carbonyl oxygens are farther than those between the K^+^ ion and the water molecules in bulk solution. It might be attributed to the fact the carbonyl oxygen atoms in the channel are generally farther from the K^+^ ion, as compared to water in its first hydration shell. In contrast, the total RDF profile (Fig. 4b, black line) and the RDF profile for the carbonyl oxygens (red line) of Na^+^ show similar trends to those in bulk, in particular, the position of the first minimum.

Subsequently, the ion coordination numbers for the ions were obtained by counting the number of carbonyl oxygens and water oxygens located in the first hydration shell when transporting through the nanochannel (Fig. 4c and d). Based on the RDF profiles in bulk, we define the radius of the first hydration shell of K^+^ and Na^+^ as 3.6 Å and 3.2 Å, respectively. The coordination number profile can reflect the dehydration and rehydration of ions during the permeation process. Initially, a fully hydrated K^+^ or Na^+^ with a coordination number of ∼6.9 or ∼5.8 approaches the entrance from the bulk. The hydration number (namely, the coordination number of water) decreased gradually to ∼2 in the nanochannel for both K^+^ and Na^+^. Meanwhile, the coordination numbers of carbonyl oxygens increase gradually to ∼6 for K^+^ and ∼4 for Na^+^, respectively, suggesting the replacement of some hydrated water by the carbonyl oxygens. It is also found that the total coordination number for K^+^ in the nanochannel is higher than that in bulk water, due to the cage-like binding manner [32]. For Na^+^, the total coordination number is nearly uniform along the nanochannel, indicating that the nanochannel accommodates Na^+^ in a similar manner to that in the bulk.

**Figure 5.**
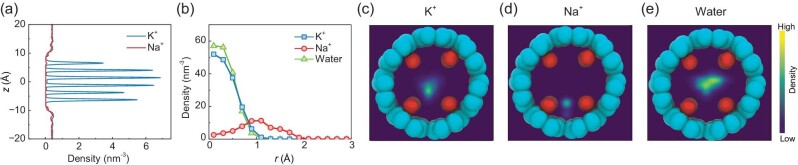
Spatial distributions of ions and water molecules under 0.2 V/nm. (a) Density profiles of K^+^ and Na^+^ along the channel axis. (b) Radial density profiles of K^+^, Na^+^ and water molecules as a function of the radial distance *r* from the channel axis. (c–e) Density maps of K^+^ (c), Na^+^ (d), and water molecules (e) in the *x–y* plane.

The spatial density of ions and water molecules was further examined, as shown in Fig. 5. Along the channel axis, six K^+^ density peaks can be observed; the middle four represent ions located at S1–S4 sites, while the remaining two represent ions located at the inlet and outlet of the nanochannel, respectively (Fig. 5a). This fact aligns with the typical K^+^ permeation process, where K^+^ shows significant residence time at each site (see Fig. 3b). We also note that the density peak for K^+^ at S2 is the highest, indicating that K^+^ is stuck at the middle of the nanochannel due to the lowest electrostatic potential at the channel center (see Fig. 1a). Different from K^+^, there is no visible Na^+^ density inside the nanochannel due to the rare permeation event. Under high electric fields, the Na^+^ density becomes visible (Fig. S3). In particular, the peak is located in the middle of binding sites, indicating that Na^+^ is located between two layers of carbonyl rings instead of in the plane of a layer of carbonyl ring, as found in previous studies [33].

We further investigated the radial density distribution of ions and water molecules within the nanochannel. As shown in Fig. 5b, K^+^ and water molecules prefer to stay at the center of the nanochannel, while Na^+^ is away from the center and closer to the wall. These observations are further confirmed by the density maps of ions and water molecules (Fig. 5c–e). K^+^ and water molecules prefer to stay closer to the central region (Fig. 5c and e), while Na^+^ shows an inclination to the nanochannel wall (Fig. 5d). Hence, when K^+^ passes through the nanochannel, it is positioned closer to the channel's center, allowing it to form a cage-like coordination structure containing ∼6 carbonyl oxygens. Na^+^, on the other hand, tends to be off-center and is coordinated by ∼4 carbonyl oxygens. A similar structural feature was also observed in KcsA channels [34].

### Effect of the number of ion binding sites

**Figure 6.**
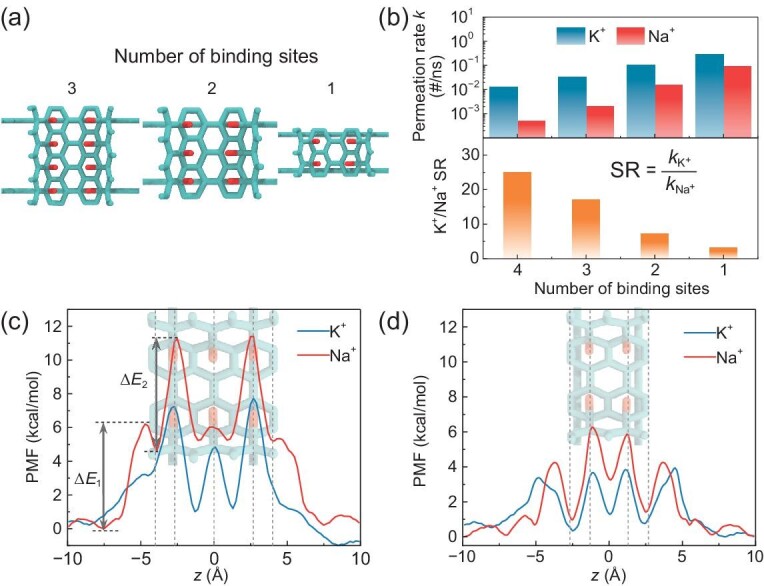
Effect of the number of ion binding sites on channel selectivity. (a) Structures of the nanochannels with three, two, and one ion binding sites. (b) Permeation rates *k* of K^+^ and Na^+^, as well as the K^+^/Na^+^ selectivity ratio (SR) in different nanochannels under 0.2 V/nm. (c and d) PMF profiles of K^+^ and Na^+^ permeating through the two-binding-site (c) and one-binding-site (d) nanochannel.

K^+^ channels are known to have remarkably high K^+^/Na^+^ selectivity due to the presence of four contiguous K^+^ binding sites in the selectivity filter. In contrast, it was also shown that a NaK channel having only two such binding sites completely loses the selectivity, despite having an overall similar selectivity filter structure as K^+^ channels [35]. Thus, it is of great interest to investigate the correlation between the number of ion binding sites and the selectivity [36,37]. To this end, we constructed a series of biomimetic nanochannels that contain fewer ion binding sites (Fig. 6a). Cumulative ion fluxes (Fig. S4) and the corresponding permeation rates were calculated. The K^+^/Na^+^ selectivity ratio was defined as the ratio of the permeation rates of K^+^ and Na^+^ (Fig. 6b). When reducing the number of ion binding sites, we find that the permeation rates for both K^+^ and Na^+^ increase significantly, and meanwhile, the K^+^/Na^+^ selectivity ratio decreases. This trend can be further understood by the one-dimensional potential of mean force (PMF) calculated from umbrella sampling simulations (see Methods for details). For the nanochannel containing two ion binding sites, it is observed that there are two energy barriers to overcome for ions to move into the nanochannel. When ions approach the entrance of the nanochannel, an energy barrier (Δ*E*_1_) of 6.2 kcal/mol and 3.1 kcal/mol was encountered by Na^+^ and K^+^, respectively (Fig. 6c). As ions move further into the first binding site, Na^+^ has to overcome an energy barrier of 6.6 kcal/mol, while a barrier of 3.5 kcal/mol is seen for K^+^ (Δ*E*_2_). In contrast, the energy barrier of Na^+^ movement between the first and second binding sites is far lower than that of K^+^, and the energy barriers for Na^+^ and K^+^ leaving the second binding site to the solution are similar to each other. These facts suggest that the selectivity is mainly attributed to the energy barrier difference at the entrance of the channel and the first binding site. As for the nanochannel with one binding site, PMF profiles show similar trends but generally lower energy barriers than those in the two-binding-site channel, consistent with the increased permeation rates of this channel (Fig. 6d). For nanochannels with three or more binding sites, multiple ions may permeate through the channel in a collective way. Therefore, multi-dimensional PMFs have to be calculated through umbrella sampling simulations; we did not do so because these calculations are computationally expensive. Based on the above findings, we expect that the K^+^/Na^+^ selectivity of these channels is still rooted in the higher energy barrier for Na^+^ than K^+^ at the entrances of the channel and the first binding site.

Finally we conducted MD simulations using a pure solution of KCl or NaCl, instead of the mixed solution used in the above simulations, to understand the influence of ion–ion interactions on the ion permeation dynamics (Fig. S5). There are two K^+^ permeation events in 300 ns simulation of the pure KCl solution (Fig. S5a). In sharp contrast, there are 12 Na^+^ permeation events in the pure NaCl solution (Fig. S5b). The difference in Na^+^ permeation rate may be related to the different coordination states of ions in the biomimetic nanochannel. In the mixed solution, the S2 site is often occupied by K^+^, which is well-coordinated by six carbonyl oxygens (see Fig. 5c) and can block the flux of Na^+^. In contrast, in the pure NaCl solution, the S2 site is occupied by Na^+^, which is off-center coordinated (see Fig. 5d) and easier to be knocked off by an incoming Na^+^, leading to a high Na^+^ flux. We also computed the PMF profiles of Na^+^ entering the nanochannel when S2 was occupied by K^+^ or Na^+^. As shown in Fig. S5c, Na^+^ has to overcome a higher energy barrier to squeeze into the binding site S4 when S2 was occupied by K^+^ as compared to Na^+^, consistent with the lower Na^+^ rate in mixed solutions.

The proposed ultra-selective CNT-based artificial potassium channels could find applications in various fields. For example, they are ideal for developing highly sensitive and selective biosensors [38], which can sieve specific ions or molecules in biological samples. These sensors are therefore useful in medical diagnostics, environmental monitoring and power generation [39]. Moreover, these artificial channels can be used as a model system to understand the transport mechanism of biological potassium channels.

## CONCLUSION

Inspired by the selectivity filter of the biological KcsA channel, we propose a computational design of a biomimetic nanochannel. Under low electric fields, the biomimetic nanochannel is highly K^+^-selective with a high permeation rate. The PMF profiles for ions passing through the nanochannel suggest that Na^+^ must overcome a higher energy barrier than K^+^ when entering the binding sites from the bulk. K^+^ and Na^+^ have distinct coordination features in the nanochannel: K^+^ is on-center coordinated at the cage sites and Na^+^ is off-center coordinated. Furthermore, when reducing the number of ion binding sites from four to one, the permeation rates of K^+^ and Na^+^ both increase but the K^+^/Na^+^ selectivity ratio decreases.

## METHODS

### Construction of the nanochannel

It is noted that the longitudinal distance of the hexatomic ring in the pristine armchair CNT is 2.45 Å, while the longitudinal distance between each layer of carbonyl rings in the crystal structure of KcsA is ∼2.9 Å [15] (Fig. 1a). Therefore, to better match the structural arrangement of carbonyl oxygens in the KcsA channel, we first stretched the pristine CNT to a stain of 10% using the LAMMPS package [40]. The AIREBO potential [41] was used to describe the interaction between carbon atoms in the CNT. We then chose the middle section of the CNT with a length of 13.34 Å as the initial model, whereby the longitudinal distance of the hexatomic ring is ∼2.7 Å.

### MD simulations

All MD simulations were performed with NAMD version 2.14 [42]. The TIP3P model [43] was used to describe water molecules, and the CHARMM36 force field [44] was used to describe ions. The carbonyl oxygens and the connected carbon atoms were described using parameters from the backbone carbonyl groups in the CHARMM36 force field. Other carbon atoms in the nanochannel, along with those in the graphene sheets, were described as sp2-like aromatic carbons (CA) in the CHARMM36 force field. The 12–6 Lennard-Jones (L-J) potential was used to describe the van der Waals interactions between different particles, with the parameters summarized in Table S1. The partial charge of each atom of the nanochannel was calculated using density function theory (DFT) method (see details in Supplementary text and results in Table S1). The cutoff distance for van der Waals interactions was set to 12 Å. The periodic boundary conditions were applied to all three directions, and the particle mesh Ewald (PME) method was utilized to treat the long-range electrostatic interactions [45]. The Langevin method was employed to maintain the temperature at 300 K [46]. A time step of 2 fs was used. After 1000 steps of energy minimization, the system was first equilibrated under the NPT ensemble for 2 ns. Subsequent production simulations from 300 to 2000 ns were conducted under the NVT ensemble. The outermost carbon atoms at both ends of the nanochannel and the two graphene sheets were fixed throughout the simulations. External electric fields were applied along the positive direction of the *z*-axis to drive ions through the nanochannel. The electrostatic potentials were computed with the PMEPot plugin [47] of the VMD package [48].

### Free energy calculations

The one-dimensional potential of mean force of K^+^ and Na^+^ passing through the nanochannel was determined with the umbrella sampling method [49]. A series of simulations were performed with a single ion being harmonically restrained in 0.5 Å step along the *z* direction with a force constant of 20 kcal/mol/Å^2^. The target position was varied from −10 to 10 Å. The starting coordinates of the two ions were arranged in a single column, aligned vertically along the central axis in the *z*-direction. Each window was run for 2 ns, with the first 1 ns being considered as equilibration. Finally, collective analysis was made with the weighted histogram analysis method (WHAM) [50], according to the implementation of Grossfield [51].

## Supplementary Material

nwae242_Supplemental_File
