# Supplementary material for: A highly-selective biomimetic potassium channel
Source: Natl Sci Rev. 2024 Jul 13;11(8):nwae242. doi: 10.1093/nsr/nwae242 (PMC11334718; doi:10.1093/nsr/nwae242)
Supplement: nwae242_Supplemental_File [file nwae242_supplemental_file.pdf]

Supplementary Data

A highly-selective biomimetic potassium channel

Junliang Zhu, Hu Qiu*, Wanlin Guo*

Key Laboratory for Intelligent Nano Materials and Devices of the Ministry of Education, State Key Laboratory of Mechanics and Control for Aerospace Structures, Institute for Frontier Science, Nanjing University of Aeronautics and Astronautics, Nanjing 210016, China

***Corresponding authors**. E-mails: qiuhu@nuaa.edu.cn; wlguo@nuaa.edu.cn

Supplementary text

Calculation of the charge distribution of the biomimetic nanochannel

We first optimized the geometric structure of the CNT nanochannel using density functional theory (DFT) in a localized Gaussian basis set (B3LYP/6-31G) via the Gaussian09 code [1]. We considered the solvent effect with water being described using the SCRF keyword along with the polarizable continuum model (PCM) [2]. The atomic charge was calculated with the CHELPG method [3]. The results showed that the charges of carbon atoms distant from carbonyl groups were close to zero, and the use of B3LYP/6-31G* basis set yielded similar results as the B3LYP/6-31G basis set (Fig. S6a-c). Therefore, in subsequent MD simulations only the atoms within three atomic intervals from the carbonyl groups were assigned with partial charges (Fig. S6d and S6e), with values obtained by averaging over equivalent positions (Table S1).

Calculation of the electrostatic potential of the KcsA channel

The crystal structure of the KcsA channel was obtained from the Protein Data Bank (1K4C) [4]. The Glu-71 residue was constructed in a protonated state, while the others were in their default states. The protein was then embedded into a POPC bilayer. The protein-lipid complex was immersed in a water solution with dimensions of 10×10×10 nm^3. K^+ and Cl^- ions were added to the system to neutralize it and reach a salt concentration of 0.15 M. The final system contained about 86,500 atoms. All the simulations were conducted with NAMD version 2.14 [5], with the CHARMM36m force fields for proteins, lipids and ions [6]. Water molecules were described with the TIP3P model [7]. Other simulation details are identical to those adopted in the main text. The system was initially minimized for 5000 steps, followed by a 2-ns equilibration simulation with a gradually decreasing harmonic constrain being applied to the protein. Finally, a 5-ns production run was conducted with all protein backbone atoms being restrained with a force constant of 2 kcal/mol/$Å^2$. Note that in all simulation a fictive wall was applied to the system in order to ensure that no ions were present in the KcsA selectivity filter. The electrostatic potentials were computed with the PMEPot plugin [8] of the VMD package [9].

Table S1: Atomic charge settings and force field parameters used in the simulation

	Atom type[a]	ε (kcal/mol)	R_{min} (Å)	Charge (e)
CNT and graphene	C_{sp2}	0.0700	3.9848	0
	C1	0.0700	3.9848	-0.35
	C2	0.1100	4.0000	0.47
	C3	0.0700	3.9848	-0.2
	C4	0.0700	3.9848	0.2
	$O_{C=O}$	0.1200	3.4000	-0.24
Ions	K^+	0.0870	3.5275	1.0
	Na^+	0.0469	2.8215	1.0
	Cl^-	0.1500	4.5400	-1.0
H_2O	O_W	0.1521	3.5364	-0.834
	H_W	0.046	0.4490	0.417

[a]All carbon atoms of the CNT and graphene were treated as C_{sp2} type, except those near carbonyl oxygens that were treated as C1, C2, C3 or C4 (see details in Fig. S6e). The $O_{C=O}$ type corresponds to the carbonyl oxygens.

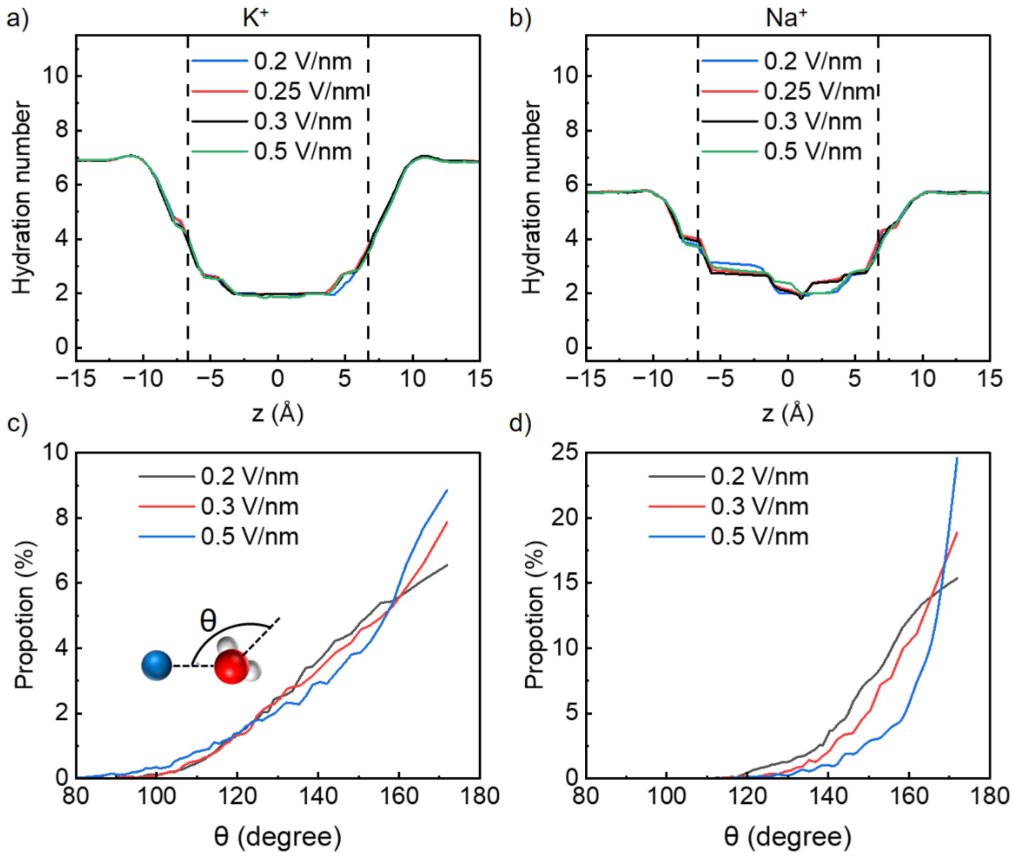

Figure S1. Influence of the electric field strength on the ion hydration properties. (a, b) The hydration number of K^+ (a) and Na^+ (b) when transporting through the nanochannel under different electric fields. The vertical dotted lines represent the position of the channel entrance. (c, d) Probability distribution of θ for water molecules in the first hydration shell of K^+ (c) and Na^+ (d) inside the nanochannel under different electric fields. Insets in panel (c) show the definition of θ.

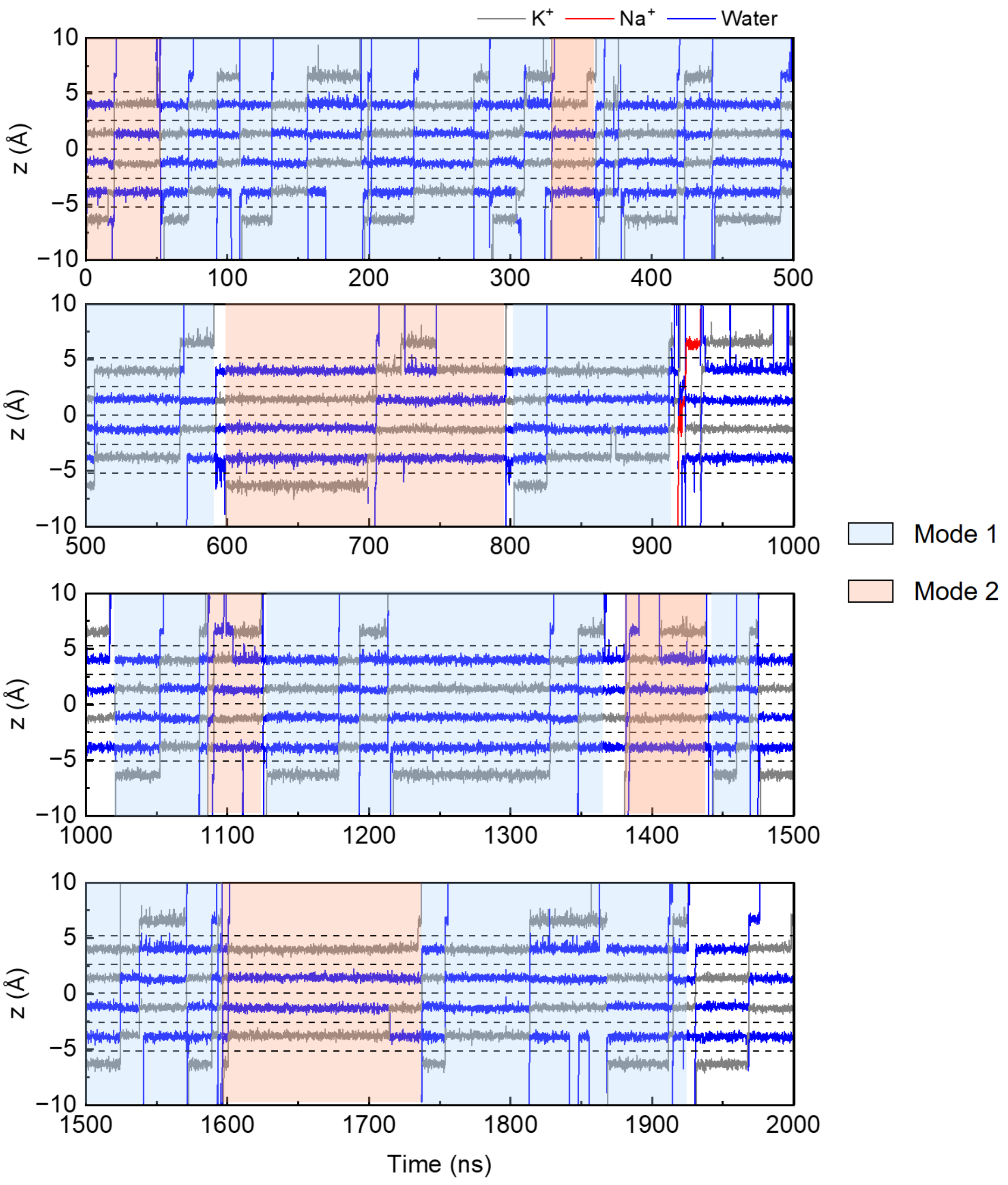

Figure S2. Detailed ion permeation dynamics through the nanochannel. The whole 2000-ns-long trajectory was divided into four intervals. The *z* coordinates of the K^+, Na^+, and water molecules are shown in black, red, and blue, respectively. The light blue blocks represent mode 1 of the permeation of K^+ and the orange one represents mode 2 of the permeation of K^+.

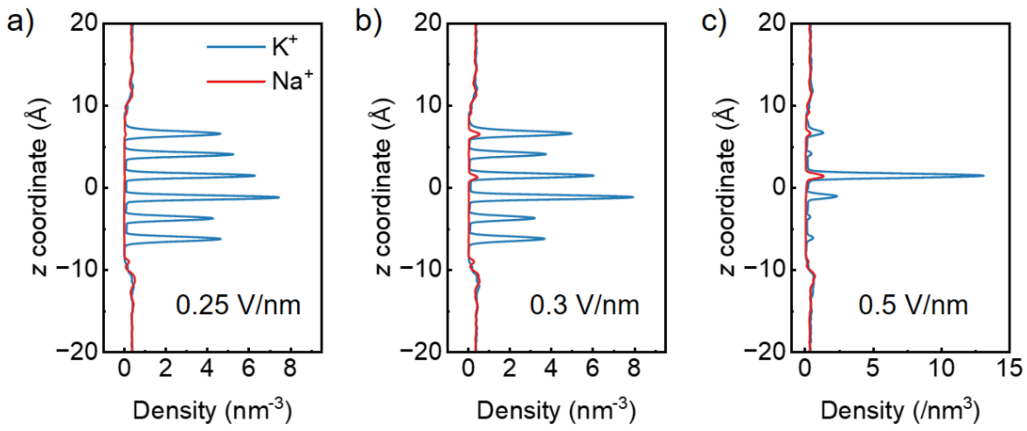

Figure S3. Density profiles for K^+ (blue line) and Na^+ (red line) in the *z*-axis under an electric field of (a) 0.25 V/nm, (b) 0.3 V/nm, (c) 0.5 V/nm.

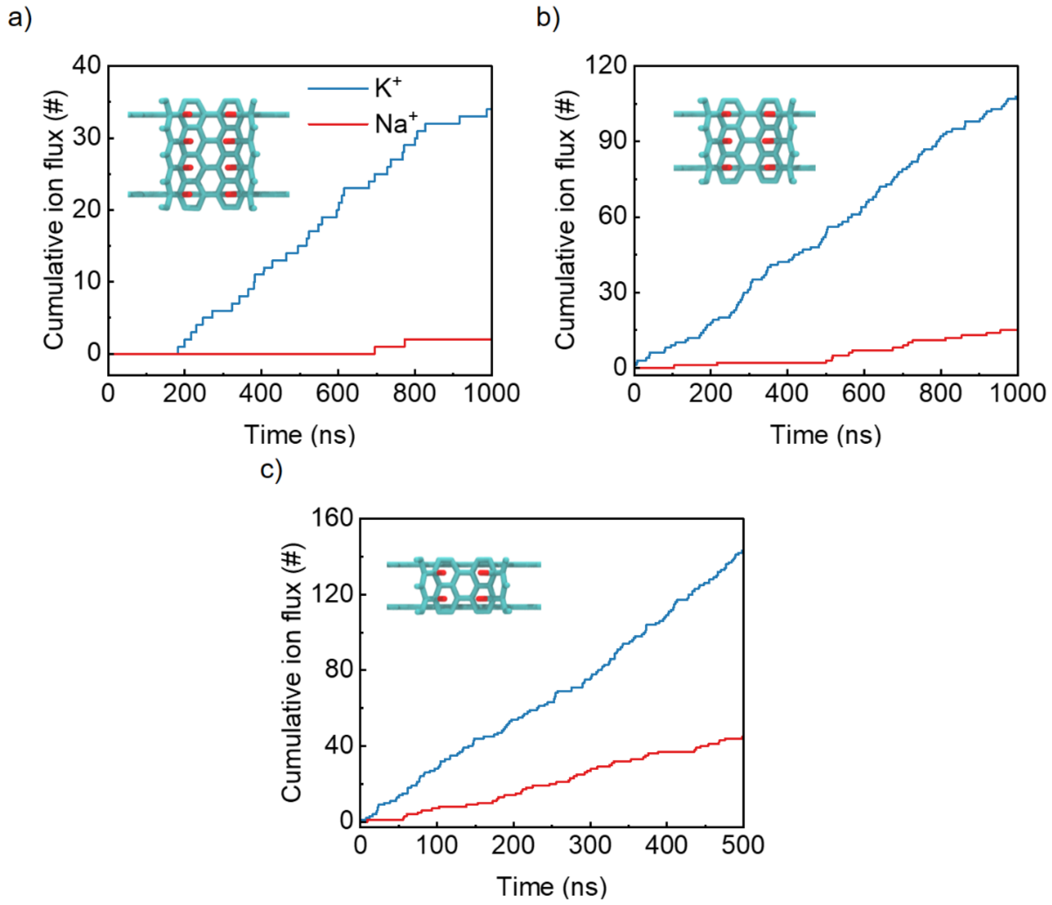

Figure S4. Cumulative ion fluxes of K^+ and Na^+ through the biomimetic nanochannel with three ion binding sites (a), two ion binding sites (b), and one ion binding site (c). The electric field used in these simulations is 0.2 V/nm.

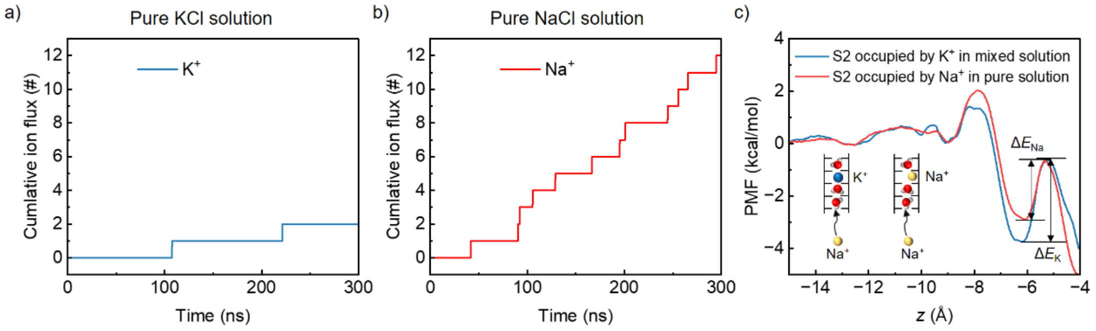

Figure S5. K$^+$ and Na$^+$ permeation in simulations with pure solutions under 0.2 V/nm. (a) Cumulative K$^+$ flux in a 0.5 M KCl solution. (b) Cumulative Na$^+$ flux in a 0.5 M NaCl solution. (c) PMF profiles of Na$^+$ entering the nanochannel with S2 occupied by K$^+$ (blue line) or Na$^+$ (red line). The insets show the schematic diagram of the calculation model. The PMF profiles were calculated using umbrella sampling.

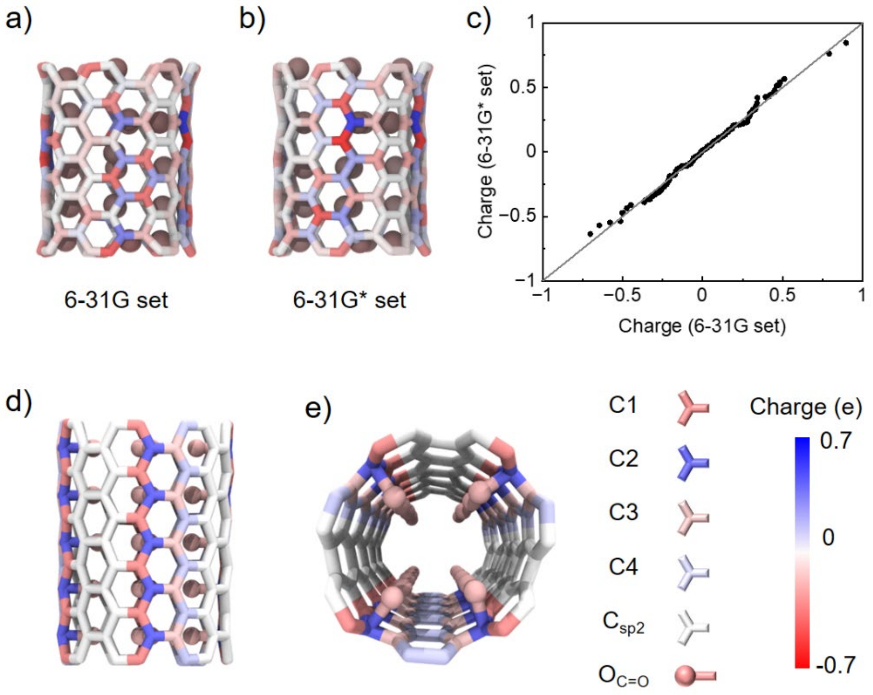

Figure S6. The calculated atomic charges of the CNT channel. (a-c) Comparison of atomic charges of the nanochannel calculated using the 6-31G and 6-31G* basis sets. (d, e) Side (d) and top views (e) of the nanochannel showing the charge distribution used in MD simulations.

References:

[1] Zheng G, Sonnenberg L, Hada M *et al*. Gaussian 09. *Gaussian Inc., Wallingford CT* 2009.

[2] Miertuš S, Scrocco E and Tomasi J. Electrostatic interaction of a solute with a continuum. A direct utilizaion of AB initio molecular potentials for the prevision of solvent effects. *Chem Phys* 1981; **55**: 117-29.

[3] Breneman CM and Wiberg KB. Determining atom-centered monopoles from molecular electrostatic potentials. The need for high sampling density in formamide conformational analysis. *J Comput Chem* 1990; **11**: 361-73.

[4] Zhou Y, Morais-Cabral JH, Kaufman A *et al*. Chemistry of ion coordination and hydration revealed by a K^+ channel–Fab complex at 2.0 Å resolution. *Nature* 2001; **414**: 43-8.

[5] Phillips JC, Hardy DJ, Maia JDC *et al*. Scalable molecular dynamics on CPU and GPU architectures with NAMD. *J Chem Phys* 2020; **153**: 044130.

[6] Huang J, Rauscher S, Nawrocki G *et al*. CHARMM36m: an improved force field for folded and intrinsically disordered proteins. *Nat Methods* 2017; **14**: 71-3.

[7] Jorgensen WL, Chandrasekhar J, Madura JD *et al*. Comparison of simple potential functions for simulating liquid water. *J Chem Phys* 1983; **79**: 926-35.

[8] Aksimentiev A and Schulten K. Imaging alpha-hemolysin with molecular dynamics: Ionic conductance, osmotic permeability, and the electrostatic potential map. *Biophys J* 2005; **88**: 3745-61.

[9] Humphrey W, Dalke A and Schulten K. VMD: visual molecular dynamics. *J Mol Graphics* 1996; **14**: 33-8.
